# Young onset type 2 diabetic patients might be more sensitive to metformin compared to late onset type 2 diabetic patients

**DOI:** 10.1038/s41598-017-16658-x

**Published:** 2017-11-27

**Authors:** Feng-fei Li, Bing-li Liu, Guo-ping Yin, Dan-feng Zhang, Xiao-fang Zhai, Mao-yuan Chen, Xiao-fei Su, Jin-dan Wu, Lei Ye, Jian-hua Ma

**Affiliations:** 1 Department of Endocrinology, Nanjing First Hospital, Nanjing Medical University, Nanjing, China; 2 National Heart Research Institute Singapore, National Heart Centre, Singapore, Singapore

## Abstract

It is unknown whether YOD (young onset diabetes) and LOD (late onset diabetes) require similar insulin doses for intensive insulin therapy with a metformin add-on to achieve glycemic control. We analyzed data from our two previously performed randomized, controlled open-label trials. Patients were randomized to receive either continuous subcutaneous insulin infusion (CSII) therapy or CSII combined with metformin therapy for 4 weeks. The studies concentrated on the differences in the insulin doses used for the two groups. We included 36 YOD (age < 40 yrs) and 152 LOD (age > 40 yrs) patients. YOD patients who received metformin combined with CSII therapy required significantly lower insulin doses to maintain euglycemic control compared to patients with LOD. A multivariate analysis, controlled for gender and the fasting blood concentration, was performed to determine the significance of the differences between groups, particularly with respect to the total and basal insulin doses. There was a trend toward improvement in β-cell function and insulin resistance in terms of ΔHOMA-B and ΔHOMA-IR in patients with YOD compared to those with LOD. Newly diagnosed T2D patients with YOD required significantly lower insulin doses, particularly basal insulin doses, to maintain glycemic control compared to the LOD patients.

## Introduction

Patients with young-onset type 2 diabetes (YOD), defined as those less than 40 years old^1,2^ at diagnosis, have more difficulty with glycemic and lipid control compared to older onset type 2 diabetes (T2D) patients: YOD patients have higher HbA1c values and LDL cholesterol^2^. Patients with YOD may thus have a higher risk of complications compared to those with T2D of later onset^3^ and T1D^4^. This has been shown to be particularly relevant for ischemic heart disease, neuropathy^5^, retinopathy^2^, and cardiovascular-renal events with YOD^6^. These findings call attention to the necessity of more attention on elucidating and defining the unique characteristics of YOD and more aggressive metabolic control^3,7^. Although some have attributed the lack of consistent treatment in younger patients^8,9^ as an important reason for fewer patients with YOD achieving an HbA1c target than patients with late-onset diabetes^2^, this has not been established at this point.

More than half of the patients with newly diagnosed T2D responded well to Continuous Subcutaneous Insulin Infusion (CSII) therapy in terms of improvements in β-cell function and glycemic control for 1 year after cessation of therapy^10^. Patients with initial poor glycemic control on multiple daily insulin injections (MDIs) who changed to CSII therapy for 6 months achieved a further reduction in HbA1c levels compared to those who were treated with MDI therapy only^11^, with their HbA1c levels remaining stable for a further 6 months^12^. Surprisingly, patients with higher baseline HbA1c levels^12–14^, particularly at levels above 9%^15^, had better improvement in their HbA1c^12^. Studies further demonstrated that the mechanisms by which CSII favors glycemic control may partly be the improvement in beta-cell function and insulin resistance^16–19^.

Metformin, an insulin-sparing diabetes agent^20^, has been established as the first-line therapy for T2D. Metformin exhibits glucose-lowering efficacy by enhancing insulin sensitivity in the peripheral tissues, such as the liver and muscle^21–24^. Studies confirmed that even subjects with T1D achieved significant improvement in glycemic control and lower insulin requirements when they received a metformin add-on to their CSII therapy^20^. Metformin combined with CSII therapy was reported to have the ability to shorten the time required to achieve euglycemic control, lower the daily insulin dose, and promote an improvement in β cell function^25^.

However, the response of patients with young-onset type 2 diabetes (YOD) to CSII therapy with or without metformin remains largely unknown. Our clinical trials [ClinicalTrials.gov, CliCTR-TRC-10001218 Registration date 13^th^ Feb. 2011, and ClinicalTrials.gov, number NCT03226210 Registration date: 19^th^ Jul. 2017] were designed to assess the glycemic variability in T2D patients who were treated with intensive insulin with or without metformin add-on. Our results (ClinicalTrials.gov, number CliCTR-TRC-10001218) showed that CSII therapy only provided a significant improvement in glycemic variation in newly diagnosed or in longstanding T2D patients^26^. In the present paper, we have analyzed the results according to age, and we report that patients with YOD were more sensitive to a metformin combination with CSII therapy in terms of requiring significantly lower insulin doses to maintain euglycemic control compared to those with a later age of onset.

## Results

A total of 188 newly diagnosed type 2 diabetic patients (95 patients received CSII in combination with metformin and 93 patients were treated with CSII therapy only) were recruited for the two studies. The enrolled subjects were divided into two groups according to whether the onset age was earlier or later than 40 years. A total of 36 (24%) patients were defined as YOD (28 M/8 F) and 152 (90 M/62 F) were defined as late onset diabetes (LOD). Patients in the YOD and LOD groups had similar C-peptide, insulin, and blood glucose levels at 0, 30, and 120 min after oral glucose loading (P > 0.05). Patients with YOD had a higher body weight (P < 0.01). There were no significant differences in β-cell function (HOMA-B and insulinogenic index, P > 0.05, respectively) and insulin resistance (HOMA-IR, Matsuda index, P > 0.05, respectively) at baseline between the two groups (Table 1).

**Table 1:** Demographic characteristics of the study subjects.

	CSII ± Met		P	CSII		P	CSII + Met		P
	YOD	LOD		YOD	LOD		YOD	LOD	
	36	152		17	76		19	76	
Age (Years)	33.4 ± 6.0	50.9 ± 5.2	0.00	34.7 ± 6.0	50.9 ± 5.4	0.00	32.4 ± 6.0	51.0 ± 5.0	0.00
Sex (M/F)	28/8	90/62	0.06	12/5	38/38	0.18	16/3	52/24	0.26
BW	78.8 ± 16.4	69.8 ± 10.3	0.00	74.3 ± 16.0	68.6 ± 10.9	0.09	83.3 ± 16.0	71.5 ± 9.3	0.00
HbA1C (%)	10.1 ± 1.9	10.3 ± 1.7	0.73	10.4 ± 1.9	10.2 ± 1.8	0.62	9.9 ± 2.0	10.4 ± 1.6	0.32
C-P_0 min_	2.5 ± 1.5	2.2 ± 0.8	0.14	2.3 ± 1.9	2.2 ± 0.9	0.70	2.6 ± 1.1	2.2 ± 0.7	0.06
C-P_120 min_	5.0 ± 2.6	5.0 ± 2.2	0.94	4.4 ± 2.8	5.6 ± 2.4	0.09	5.4 (3.7,6.7)	4.3 (3.0, 5.5)	0.05
INS_0 min_	8.67 ± 8.17	7.3 ± 5.0	0.21	7.6 ± 8.5	6.9 ± 5.8	0.68	9.5 ± 8.0	7.7 ± 4.0	0.18
INS_120 min_	23.43 ± 19.1	23.0 ± 17.8	0.90	17.9 ± 17.3	26.1 ± 21.4	0.17	21.3 (14.6,33.4)	17.6 (10.5,26.6)	0.13
Glu_0 min_	10.95 ± 3.46	11.3 ± 2.7	0.57	11.5 ± 3.7	11.0 ± 2.5	0.50	10.5 ± 3.2	11.5 ± 2.8	0.16
Glu_120 min_	21.6 ± 4.8	22.3 ± 4.0	0.34	22.1 ± 5.4	23.1 ± 4.0	0.42	21.1 ± 4.3	21.5 ± 3.8	0.72
HOMA-β	3.9 ± 3.3	3.5 ± 2.5	0.52	2.9 ± 2.0	2.9 ± 2.1	0.93	4.7 ± 4.0	4.4 ± 2.8	0.68
HOMA-IR	19.9(11.2,32.8)	16.6 (10.8,26.8)	0.41	40.9 ± 100.3	21.9 ± 24.5	0.17	24.3 (13.3,50.1)	17.0 (12.3,26.5)	0.12
MI	6.4 ± 6.3	5.8 ± 4.3	0.54	8.2 ± 7.9	6.5 ± 5.1	0.29	4.8 ± 3.8	5.0 ± 2.7	0.80
II	11.7 ± 16.2	8.6 ± 10.6	0.20	11.2 ± 15.6	8.2 ± 8.0	0.29	12.1 ± 17.2	9.0 ± 13.3	0.44

Data were presented as the means ± SD or IQR. CSII: Continuous subcutaneous insulin infusion, YOD group: Young onset diabetes, LOD group: Old onset diabetes, M/F: Male/female, BW: Body weight (Kg), C-P_0 min:_ C-peptide concentration at 0 min after glucose loading (ng/mL), C-P_120 min:_ C-peptide concentration at 120 min after glucose loading (ng/mL), INS_0 min:_ Insulin concentration at 0 min after glucose loading (mU/L), INS_120 min:_ Insulin concentration at 120 min after glucose loading (mU/L), Glu_0 min:_ Glucose concentration at 0 min after glucose loading (mmol/L), Glu _120 min:_ Glucose concentration at 120 min after glucose loading (mmol/L), HOMA-B: Homeostasis model assessment-B, HOMA-IR: Homeostasis model assessment-IR, MI: Matsuda Index, II: Insulinogenic Index.

**Figure 1:**
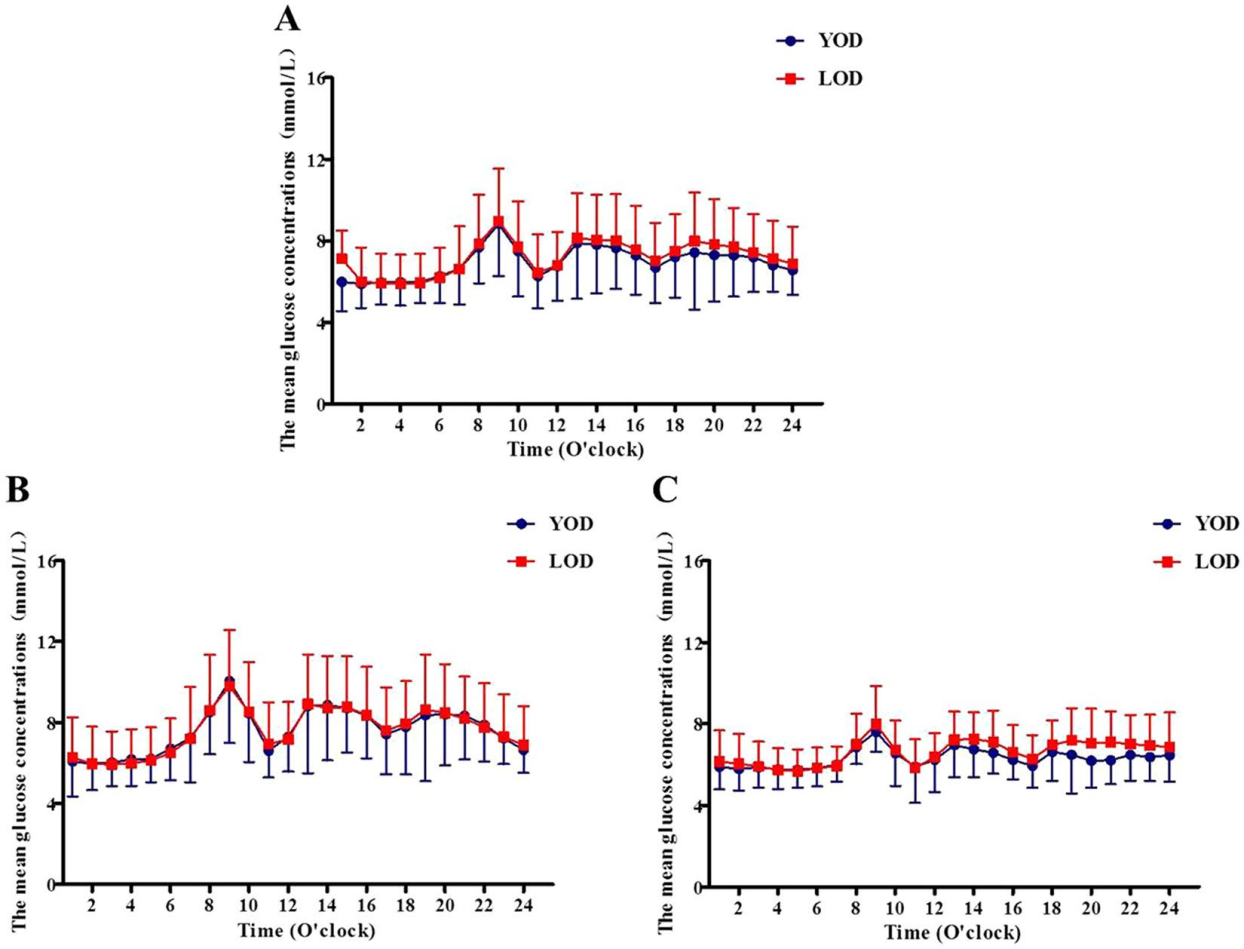
The hourly glucose concentrations calculated from CGM. The hourly glucose concentrations in all study subjects (**A**), in CSII therapy only patients (**B**) and in CSII ± Met therapy subjects (**C**).

In this current study, subjects with YOD achieved euglycemic control in 4.8 ± 2.4 days, which was similar to patients with LOD (5.1 ± 2.3, P > 0.05). We analyzed the insulin doses required by patients to maintain glycemic control after achievement of euglycemia control. Our data showed that the total, basal and bolus insulin doses were similar in the two groups (0.45 ± 0.27 vs. 0.51 ± 0.24, 0.25 ± 0.17 vs. 0.27 ± 0.13, and 0.22 ± 0.14 vs. 0.24 ± 0.16 U/Kg/day, P > 0.05, respectively). Moreover, the CGM data showed that the patients in the two groups had similar mean glucose levels per hour (Fig. 1A) and similar glycemic variations in terms of 24-hrs MBG, SD, CV%, MAGE, the time spent on and the incremental AUC of glucose concentration >10 mmol/L (P > 0.05, respectively) (Table 2).

**Table 2:** Glycemic variations monitored by CGM in the study subjects.

	CSII ± Met		P	CSII		P	CSII + Met		P
	YOD	LOD		YOD	LOD		YOD	LOD	
	36	152		17	76		19	76	
MBG	6.9 ± 1.3	7.1 ± 1.4	0.35	7.6 ± 1.3	7.6 ± 1.5	0.92	6.2 ± 0.9	6.6 ± 0.9	0.15
SD	1.4 ± 0.8	1.6 ± 0.7	0.16	1.9 ± 0.7	1.8 ± 0.7	0.78	0.9 ± 0.4	1.3 ± 0.5	0.01*
CV	0.2 ± 0.1	0.2 ± 0.1	0.10	0.2 ± 0.1	0.2 ± 0.1	0.89	0.2 ± 0.1	0.2 ± 0.1	0.01*
MAGE	3.8 ± 2.2	4.1 ± 2.0	0.40	4.9 ± 2.3	4.7 ± 2.0	0.73	2.7 ± 1.5	3.4 ± 1.7	0.11
>10 time	8.3 ± 12.5	8.9 ± 13.0	0.79	14.8 ± 14.0	13.9 ± 15.2	0.81	1.9 ± 6.0	3.1 ± 5.6	0.41
>10 AUC	0.2 ± 0.3	0.2 ± 0.4	0.95	0.3 ± 0.4	0.3 ± 0.5	0.71	0.0 ± 0.1	0.0 ± 0.1	0.48

Data were presented as the means ± SD. CSII ± Met: Continuous subcutaneous insulin infusion ± Metformin, YOD group: Young onset diabetes, LOD group: Old onset diabetes, MBG: mean glucose concentration (mmol/L), SD: standard deviation (mmol/L), CV: coefficient of variation (%), MAGE: mean amplitude of glycemic excursions (mmol/L), >10 time: the time spend on glucose concentrations above 10.0 mmol/L, >10 AUC: the incremental area under the curve of glucose concentrations above 10.0 mmol/L (mmol/L per day).

We also did not observe any differences in total, basal and bolus insulin doses (0.61 ± 0.30 vs. 0.59 ± 0.28, 0.33 ± 0.20 vs. 0.30 ± 0.15, and 0.28 ± 0.16 vs. 0.29 ± 0.20 U/Kg/day, P > 0.05, respectively), mean glucose levels per hour (Fig. 1B) and glycemic variations (Table 2) in YOD and LOD patients who received CSII therapy only.

Anti-diabetic drugs such as metformin combined with insulin can significantly reduce the glucose level in diabetics. As expected, our data showed that patients who received the metformin add-on therapy required significantly lower insulin doses in terms of total, basal and bolus insulin doses (0.32 ± 0.15 vs. 0.61 ± 0.30, 0.18 ± 0.09 vs. 0.33 ± 0.20, and 0.15 ± 0.06 vs. 0.28 ± 0.16 U/Kg/day, P < 0.01, respectively) to maintain similar glycemic control compared to those who received insulin therapy alone.

Notably, patients with YOD treated with metformin combination therapy required significantly lower total insulin doses (0.32 ± 0.15 vs. 0.43 ± 0.15 U/Kg/day, P < 0.05) compared to those with LOD to maintain glycemic control in terms of mean glucose levels per hour (Fig. 1C) and 24-hrs MBG, MAGE, the time spend on and the incremental AUC of glucose concentrations above 10 mmol/L (P > 0.05, respectively) (Table 2). Most importantly, YOD patients who were administered metformin combined with CSII showed significant improvements in glycemic variations in terms of SD and CV compared to those of LOD patients (Table 2). The total daily insulin doses were given in two modes: a bolus dose before each meal and a basal dose throughout 24 h. No differences were observed in the bolus doses administered to the two groups (0.15 ± 0.06 vs. 0.19 ± 0.07 U/Kg/day, P > 0.05). However, patients with LOD required significantly lower basal insulin doses to maintain euglycemic control compared to the patients with LOD (0.18 ± 0.09 vs. 0.24 ± 0.09 U/Kg/day, P < 0.05, respectively). In the logistic analysis, only gender and fasting blood glucose concentrations were significantly correlated with insulin doses. A multivariate analysis, controlled for gender and fasting blood concentration, was performed to determine the significance of the differences between groups, particularly with respect to the total and basal insulin doses. Our data showed that patients with YOD required significantly lower total and basal insulin doses compared to those with LOD. To identify the characteristics associated with the decreased insulin doses required by patients with YOD, we compared the baseline data of the two groups. A total of 95 patients (19 with YOD and 76 with LOD) received the metformin add-on therapy. The YOD and LOD patients had similar C-peptide, insulin, and blood glucose levels at 0 and 120 min after oral glucose loading (P > 0.05, respectively), but were dissimilar for age and body weight (P < 0.01, respectively) (Table 1).

We further compared β-cell function and insulin resistance between the groups. Our data showed that there were no significant differences in terms of HOMA-B, insulinogenic index, HOMA-IR, and Matsuda index at baseline (Table 1). After therapy, the YOD and LOD patients still had similar C-peptide, insulin, and blood glucose levels at 0 and 120 min after oral glucose loading, and similar HOMA-B, insulinogenic index, HOMA-IR, and Matsuda index values (P > 0.05, respectively).

We next analyzed changes of β-cell function and insulin resistance before and after therapy. The data indicated a tendency toward improvement in β-cell function and insulin resistance in terms of ΔHOMA-B [23.3 (0.6, 50.4) vs. 14.3 (6.6, 26.7)] and ΔHOMA-IR [−2.9 (−4.1, −0.3) vs. −1.7 (−2.6, −0.3)] in patients with YOD treated with the metformin combination therapy compared to those with LOD, but this tendency was not statistically significant. The improvement in β-cell function and decreased insulin resistance, although not statistically significant, might be attributed to the possibility that patients with YOD might be sensitive to metformin when insulin doses are lowered to maintain glycemic control.

## Discussion

The data of this current study indicated that newly diagnosed T2D patients with YOD had a significant increase in metformin sensitivity, which manifested itself when lowering insulin doses to maintain glycemic control compared to those with a later age of onset of diabetes. We also observed some improvements in β-cell function and ameliorated insulin resistance in terms of ΔHOMA-B and ΔHOMA-IR in patients with YOD compared to those with LOD. The observed improvement in β-cell function and insulin resistance might be the reason that patients with YOD might be sensitive to metformin when it is added to maintain glycemic control.

We consecutively recruited 188 subjects from February 2010 to June 2016. A total of 36 patients were diagnosed before 40 years of age, corresponding to a YOD incidence of (24%), which was consistent with findings from a previous study, with an enrolment of 41029 patients and one in five adult T2D patients with YOD^2^. Previous studies have also focused on the glycemic and lipid metabolism in T2D patients with onset earlier than 25^7^ and ≤45 years of age^27^. Those results showed that patients with YOD onset had the prominent problem of poor glycemic and lipid control and a higher prevalence of diabetic complications^5–7,27^. Various characteristics of T2D patients with YOD were reported to be associated with poor glycemic control, including younger age^2,28^, longer disease duration^28^, and genetic, socioeconomic, or psychological-behavioral factors^2^. Additional observations also indicated the importance of poor self-management resulting from chronic stress and negative life events^29^. Smoking and alcohol intake may also contribute to the poor glycemic and lipid control in patients with YOD^2^. Compared to T2D patients diagnosed at a later age, fewer YOD patients achieved HbA1c levels of <7%^2^. It is significant to note that T2D patients with an HbA1c level above 6.5% had a higher death rate than those with an HbA1c concentration below 5.7%^30^. This gives validity to the notion that optimal treatment aimed at improving glycemic control in this high-risk group of diabetic patients is of paramount importance.

Drug-naïve patients with T2D responded well to Continuous Subcutaneous Insulin Infusion (CSII) therapy in terms of improvement in β-cell function and glycemic control. Treatment has shown that half of those patients could maintain euglycemic control for 1 year without any additional glucose-lowering agents^10^. Patients with poor glycemic control and HbA1c concentrations above 8% after pre-randomization with multiple daily insulin injections (MDIs) can achieve a significant reduction in HbA1c levels^11^, and their HbA1c levels can remain stable for 12 months with CSII therapy^12^. A stratified analysis comparing the baseline characteristics revealed that patients with an HbA1c level above 9% were proven to have favorable outcomes associated with the decrease in HbA1c^15^. In this study, patients in the two groups had HbA1c levels above 10% and similar diabetes duration, β-cell function, and insulin resistance; however, they differed in age and body mass index (BMI). Some patients with different ages, diabetes durations, complications, BMI, insulin resistance^31^, and different β-cell function or C-peptide levels^32^ benefit from reductions in their HbA1c levels. Thus, they should respond similarly to the CSII combination with metformin therapy.

Our data showed that T2D patients with YOD required significantly lower insulin doses, particularly basal insulin doses, to maintain glycemic control compared to those with a later onset after 40 years of age in the metformin and CSII therapy group. However, we did not identify any differences in insulin doses in patients with only CSII therapy in the two groups. Those results indicated that T2D patients with YOD were likely to benefit the most from metformin, in view of its demonstrated benefits as a glucose-lowering agent to enhance insulin sensitivity^21–24^. Metformin has already been recommended as the first-line glucose-lowering agent in the management of T2D in most guidelines. In combination with insulin in the treatment of T2D, the use of metformin leads to an increase in insulin sensitivity and the reduction of daily insulin doses^21–23^. However, we did not see statistically significant differences in insulin resistance, such as HOMA-IR, Matsuda Index, and Insulinogenic Index at baseline and after therapy between young and older patients who received metformin in addition to CSII therapy. There was some variation in the changes in improvements in β-cell function and insulin resistance in terms of ΔHOMA-B and ΔHOMA-IR from baseline to endpoint in patients with YOD compared to those with LOD. A limitation should be noted: in this study, we did not adequately explain the significantly lower insulin doses that the younger patients with T2D required to maintenance euglycemic control compared to those of the older patients. In our study population, there were significantly more male than female YOD patients. Although sex hormones can affect glucose metabolism and insulin sensitivity, in the LOD patients, there was no difference between male and female patients. Our study did not have sufficient power to distinguish between the YOD male and female patients adequately. Future investigation of the reasons behind treatment by age is necessary to address this important difference.

Patients with high glycemic variations may not have favorable outcomes with CSII therapy^31^ in terms of improvements in glycemic control, and they may require a CSII in combination with another glucose-lowering agent therapy^33^. In this study, we did not observe the efficacy of metformin combined with CSII therapy, which resulted in changes of HbA1c concentrations. HbA1c is an important glycemic control index in large-scale clinical trials^34–36^, and the association between the reduction in HbA1c levels and the reduction in the risks of diabetic complications has been well established^37^. We previously observed that patients with higher HbA1c levels may have a larger MAGE compared to those with a lower HbA1c level^38^. However, patients with similar HbA1c values do not exhibit the same glycemic variations^39–41^. Our CGM data showed that patients who received metformin add-on insulin therapy had significantly improved glycemic variations in terms of MAGE, SD, CV%, and time spent on and the incremental AUC of glucose concentrations ≥10 mmol/L, which might be significant in avoiding long-term diabetic complications. The drastic blood glucose concentrations may lead to an over-production of peroxynitrite and nitrotyrosine^42–44^, which may be an indicator of diabetic vascular complications^45^.

In conclusion, our data revealed that newly diagnosed T2D patients with YOD required significantly lower insulin doses, particularly basal insulin doses, to maintain glycemic control compared to LOD patients. Our data indicated that patients with YOD responded well to metformin combined with CSII treatment in terms of lowering the insulin doses necessary to improve β-cell function and alleviate insulin sensitivity.

## Materials and Methods

The two studies were both randomized, controlled open-label trials. Study protocols and patient consent forms were approved by the Institutional Ethical Committee of Nanjing First Hospital and the institutional Ethical Committees of the other centers. All patients gave written informed consent to participate. The methods were performed in accordance with the Declaration of Helsinki guidelines, including any relevant details.

This study (CliCTR-TRC-10001218) was a multiple-center, randomized, parallel-group trial, which including a 4–6 day run-in period of diet alone and a 2–3-week randomized phase as previously described^26^. In the initial testing, T2D patients aged 18–80 years who had an HbA1c value ranging from 9.0% to 12.0% were enrolled from eight centers in China between February 2010 and December 2014. All patients who were admitted as inpatients received intensive insulin therapy without any oral glucose-lowering agent. The other study (NCT03226210) was as a single-center, randomized, controlled open-label study conducted between April 2012 and June 2016. Newly diagnosed T2D cases aged between >18 and <80 years and with HbA1c values >9.0% at diagnosis were recruited. Patients were excluded from the analysis if they had serum creatinine levels ≥1.5 mg/dL (males), ≥1.4 mg/dL (females) or abnormal creatinine clearance, known as hypersensitivity to metformin or insulin. Patients with infection or acute metabolic diabetic complications such as ketoacidosis or hyperosmolar state (coma) and patients who used systemic glucocorticoids in the last 3 months were excluded. If the patients were unable to tolerate the minimum metformin dose (1000 mg/day), they were also excluded from this study. The trial included a screening period to collect baseline parameter values, a 2 ± week treatment period, and a 4-day CGM period. After screening, the enrolled subjects then received metformin add-on CSII therapy. Metformin (Bristol-Myers Squibb, USA) was administered at a dose of 500 mg thrice-daily. The total daily insulin (Aspart, Novo Nordisk, Bagsværd, Denmark) doses were 0.4 IU/kg, which was given employing a two-injection mode: 1/2 of the total daily dose was equally given as boluses before each meal, and the remaining insulin was given as a basal dose.

The two trials were conducted using the same protocol. After completing the baseline assessment and the 3-day CGM^46^, all patients were subjected to oral glucose tolerance tests (OGTTs). Serum glucose, insulin, and C-peptide concentrations at 0 and 120 min after glucose loading and HbA1c values were measured at the Department of Endocrinology, Nanjing First Hospital, Nanjing Medical University, China. All patients were subjected to retrospective CGM for 3 days (Sof-sensor, CGMS-Gold, Medtronic Incorporated, Northridge, USA) in the hospital by a specialist nurse at baseline and at endpoint^26^. Insulin doses were subsequently administered by the treating physician according to the blood glucose values obtained by self-monitoring. When euglycemic control was achieved, defined as 80% of fasting blood glucose and 2 h postprandial after each meal was less than 6.1 and 8.0 mmol/L, respectively^10^, the total daily, basal, and bolus insulin doses were recorded. The glycemic variation parameters, such as the 24 h mean amplitude of glycemic excursions (MAGE), the 24 h mean blood glucose (MBG), the standard deviation (SD) of the MBG, the percentage time duration (%) and the incremental area under curve (AUC) of glucose levels >10.0 mmol/L and <3.9 mmol/L, were calculated and recorded. β-cell function was assessed by the homoeostasis model assessment B (HOMA-B), insulin sensitivity was indicated by HOMA-IR^10,47^ and the Matsuda index was calculated as previously described^48,49^.

In this paper, we divided all newly diagnosed T2D cases receiving intensive insulin therapy, with or without metformin, into two groups based on those aged younger than 40 or above 40 years old. The primary endpoint was the between-group differences in insulin doses. The secondary endpoints were the MBG, SD, CV, MAGE, 24-h MBG, and the time spent on and the incremental AUC of hyperglycemia (defined as sensor glucose values >10 mmol/L).

### Statistical analysis

The analyses were performed using the SPSS 16.0 (SPSS, Science, Chicago, USA) statistical package. All variables were tested for normal distribution of the data. Data are presented as the means ± SD or as the median (IQR). A binary logistic regression and Pearson analysis (Spearman’s analysis in non-parametric variables) were performed to identify the parameters likely correlated with the change of values in terms of the insulin doses. The efficacy of CSII was analyzed by the paired t-test or Wilcoxon test, whereas differences between the groups were examined using the student’s unpaired t-test or the Mann-Whitney U test. A two-way ANOVA for repeated measurements was used in the comparison of groups. Bonferroni’s correction was followed. All comparisons were 2-sided at the 5% significance level. A P value <0.05 was considered statistically significant.
